# Droplet-vitrification of *Aranda* Broga Blue orchid: Role of ascorbic acid on the antioxidant system and genetic fidelity assessments via RAPD and SCoT markers

**DOI:** 10.1016/j.btre.2020.e00448

**Published:** 2020-04-21

**Authors:** Soo Ping Khor, Lit Chow Yeow, Ranjetta Poobathy, Rahmad Zakaria, Bee Lynn Chew, Sreeramanan Subramaniam

**Affiliations:** a School of Biological Sciences, Universiti Sains Malaysia (USM), 11800, Gelugor, Penang, Malaysia; b School of Biological Sciences, Quest International University, 30250, Ipoh, Perak, Malaysia; c Centre for Chemical Biology, Universiti Sains Malaysia, Bayan Lepas, Penang, Malaysia

**Keywords:** APX, Ascorbate peroxidase, CAT, Catalase, Fe, Na-EDTA Ethylenediaminetetraacetic acid, Ferric Sodium, LN, Liquid Nitrogen, PLB, Protocorm-like body, RAPD, Random Amplified Polymorphic DNA, SCoT, Start Codon Targeted, SOD, Superoxide Dismutase, *Aranda* Broga Blue orchid, Cryopreservation, Antioxidant enzymes, Molecular markers, Genetic fidelity

## Abstract

A droplet-vitrification cryopreservation protocol has been successfully developed for *Aranda* Broga Blue orchid hybrid using protocorm-like bodies (PLBs). However, maximum growth regeneration percentage was recorded at 5% only based on previous report. Thus, to improve growth recovery of cryopreserved PLBs, cryopreservation stages were supplemented with ascorbic acid, tested at 50, 100 and 150 mg/L. However, results demonstrated that exogenous ascorbic acid was not favorable in regeneration of cryopreserved explants (maximum value of 1.67 % with 50 mg/L ascorbic acid supplementation). Total soluble protein and various antioxidant enzyme activities such as catalase (CAT), superoxide dismutase (SOD) and ascorbate peroxidase (APX) were evaluated after each cryopreservation stages in conjunction with the application of exogenous ascorbic acid. Addition of antioxidant must be carefully evaluated and its application may not guarantee successful growth recovery. RAPD and SCoT molecular analysis confirmed the genetic stability of regenerated cryopreserved PLBs as no polymorphism was detected compared to control PLBs culture.

## Highlights

• Exogenous ascorbic acid does not guarantee improvement of growth recovery in cryopreserved PLBs.
• Exogenous ascorbic acid and cryopreservation stages has a significant interaction effect to total soluble protein content, SOD and APX activities.
• Changes in total protein content, SOD and CAT activities do not correlated with growth recovery of cryopreserved PLBs.
• High APX activities are associated with growth recovery of cryopreserved explants.
• Genetic stability of regenerants recovered from cryopreserved *Aranda* PLBs was confirmed based on RAPD and SCoT molecular markers.

## 1 Introduction

**Fig. 1:**
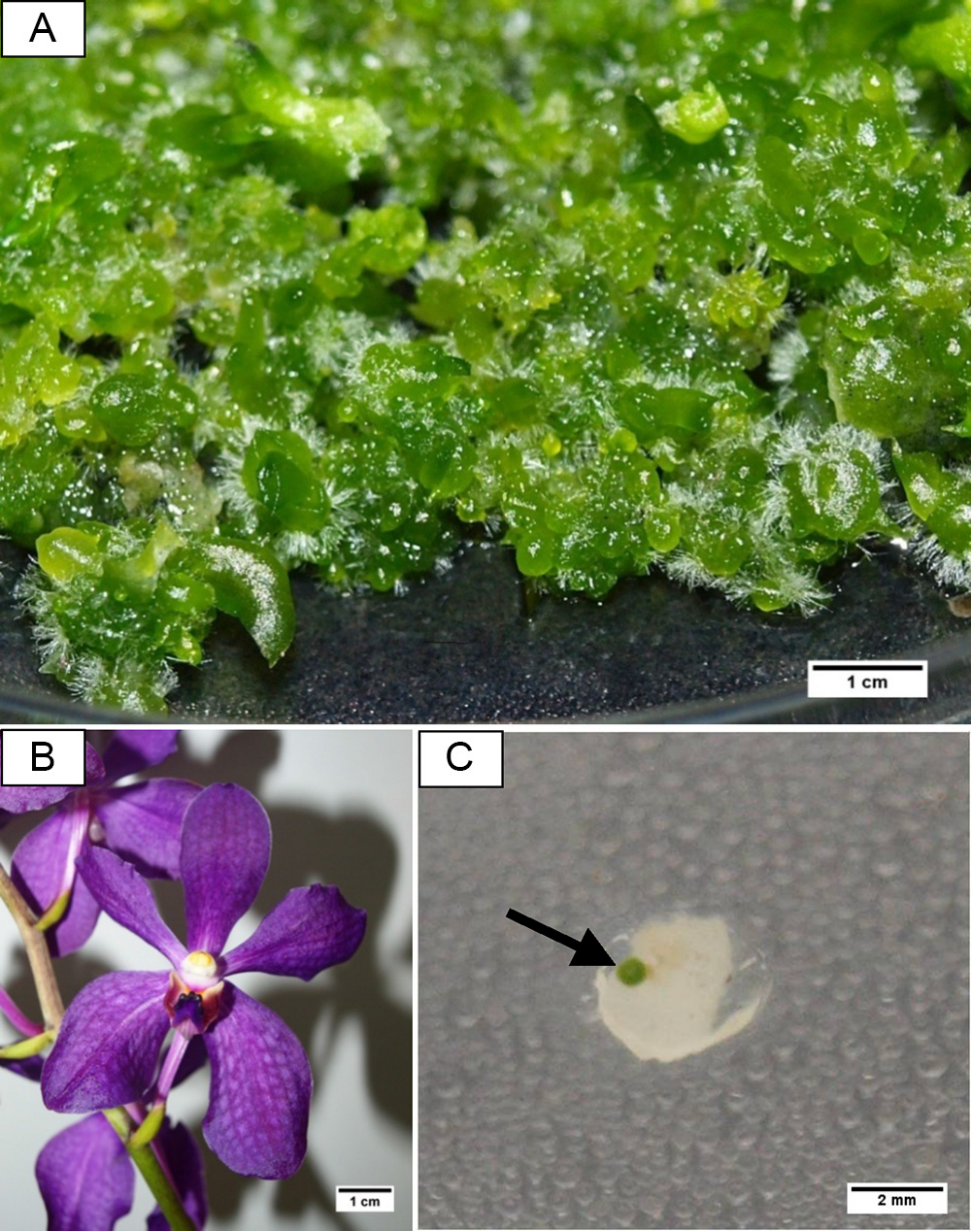
The orchid hybrid *Aranda* Broga Blue: Four(4) weeks old orchid PLBs (A), Inflorescence (B), Newly regenerated PLB (arrow pointed) observed on cryopreserved PLB after 3 weeks of growth recovery (C) (For interpretation of the references to colour in this figure legend, the reader is referred to the web version of this article).

*Aranda* Broga Blue is an orchid hydrid that is locally hybridized in Malaysia for commercialization purposes in the orchid cut flower industry [1,2] (Fig. 1B). This orchid is known to have commercial potential due to one of its parent *Vanda coerulea* that is important in orchid breeding [3] as well as rich in antioxidant and beneficial secondary metabolites [4]. Thus, a droplet-vitrification cryopreservation protocol has been successfully developed in the earlier studies in order to conserve all the important features of this orchid [2].

The effectiveness and simplicity of droplet-vitrification technique has broadened its application in various plant species cryopreservation. The theory behind this technique is the application of ultra rapid cooling and warming rate during cryopreservation process in order to prevent intracellular ice nucleation development. Under ultra rapid cooling rate, even remaining water in the cell can be vitrified and form a metastable glass instead of ice crystals. Popova et al. [5] highlighted that successful cryopreservation of partially dehydrated explants such as protcorms and PLBs could be attributed to rapid cooling and rewarming. Hence, droplet-vitrification was employed and several studies reported that high rapid and cooling rate improves the growth recovery of cryopreserved protocorms or PLBs compared to other vitrification based method [6,7]. Until recently, droplet-vitrification technique has been widely applicable in conserving commercially important crops, fruits and ornamental plants such as rose [8], taro [9], potatoes [10,11], apple [12] and orchids [7,13].

The plant tissues undergo various stresses throughout cryopreservation process during excision of explants, osmotic dehydration and extreme temperature changes [14,15]. These stresses imposed on plant tissues results in cell damage, subsequently leads to reduced growth regeneration or cell death upon thawing [14]. Reactive oxygen species (ROS) mediated oxidative stress has been known as the fundamental factor that caused cell damage in cryopreserved explants [16]. Ascorbic acid (Vitamin C) is a well known antioxidant and it has been widely used in cryopreservation protocol with improvement of regeneration obtained in cryopreserved plants such as blackberry [14] and *Nephelium ramboutan-ake* shoot tips [17]. Uchendu et al. [18] recommended that it is essential to include exogenous antioxidants as part of the standard procedure during plant cryopreservation. As a consequence, it is required to access the effects of exogenous antioxidants to plant defence mechanism against oxidative stresses throughout cryopreservation treatment. By revealing the plant defence response and mechanism of abiotic stress arises from cryoprotectants and pre-cryostorage treatment, this will help to improve plant cell survival after cryogenic treatment [19]. This assessment is thought to be particularly important in highly recalcitrant and freezing sensitive plant species.

The successful cryopreservation process should guarantee the regeneration of genetically identical and true to type cryopreserved materials. Development of genetic variation assessment technique for cryopreserved explants is an utmost important study in cryopreservation research. Random amplified polymorphic DNA (RAPD) utilizes only a short arbitrary primer (10 nucleotides) to amplify random segment of DNA that is complementary to it and it was known that RAPD system yields high level of polymorphism [20]. Gene targeted marker such as Start codon targeted SCoT marker based on the short conserved region of plant genes surrounding the ATG start codon, was design by Collard and Mackill [21]. Due to the basis of the design from consensus sequence of the plant genes, the SCoT markers that generated may be correlated to functional genes and their corresponding traits [21,22].

The aim of this study attempted to determine if exogenous ascorbic acid is essential in cryopreservation protocol and its effects to antioxidant enzyme activities of cryopreserved *Aranda* Broga Blue protocorm – like bodies (PLBs) were evaluated. The present study also attempt to explore the possible role of antioxidant enzymes to cryoprotective mechanism of tropical plant species. Successfully regenerated cryopreserved PLBs were also subjected to RAPD and SCoT molecular analysis to examine their genetic stability. Up to date, there has been no report attempting to apply SCoT marker on *Aranda* genus orchid.

## 2 Materials and methods

### 2.1 Micropropagation of *Aranda* Broga Blue PLBs

The PLBs were aseptically cultured in Vacin and Went (VW) semi-solid basal medium [23] supplemented with 2 % (w/v) banana homogenate, 2 % (w/v) potato, 15 % (v/v) fresh coconut water, 1 g/L activated charcoal (Duchefa, Netherlands) and 4.8 g/L Gelrite™ (Duchefa, Netherlands) (referred as VW100) media as reported by Khor et al. [2] (Fig. 1A). Micropropagation of PLBs was performed every 4–6 weeks and grown at 25 ± 2 °C incubation temperature, under 150 μmol m^-2^s^-1^ cool white fluorescent lamps (Philips TL-D, 36 W) with 16 h photoperiod. Four (4) weeks old PLBs (3−4 mm) was selected as plant material in the following cryopreservation experiments.

### 2.2 Effects of exogenous ascorbic acid in four critical droplet-vitrification cryopreservation stages

Cryopreservation of *Aranda* Broga Blue PLBs was conducted according to the protocol demonstrated by Khor et al. [2]. Four (4) weeks old PLBs (3−4 mm) were precultured on semi-solid preculture media containing 0.2 M sucrose for 3 days and incubated at standard tissue culture incubation temperature (25 °C). Next, PLBs were treated with 1.5 mL of loading solution [24] for 20 min at 25 °C, followed by immersed into 1.5 mL of Plant vitrification solution 2 (PVS2) at 0 °C for 20 min [25]. Each PLB were then transferred to a droplet of PVS2 (20 μL) aliquot on a piece of aluminium foil (10 mm x 30 mm) (Diamond Heavy Duty Aluminium Foil, Reynolds Consumer Products Inc. USA), 5 min before the vitrification treatment ends. The aluminium foils were then immediately immersed in liquid nitrogen (LN) and maintain for at least 30 min. Next, cryopreserved PLBs were thawed in 10 mL unloading solution [58] at 25 °C and were left incubated for 20 min. LN storage step was excluded for non-cryopreserved PLBs. Growth recovery of PLBs was performed on VW10 medium [2] containing 0.2 % (w/v) banana homogenate, 0.2 % (w/v) potato, 1.5 % (v/v) fresh coconut water and 4.8 g/L Gelrite™ with a piece of filter paper lined on top. PLBs were maintained in complete darkness for 24 h, followed by transferring to fresh VW10 medium and incubate in complete darkness for the next 1 week. PLBs were then exposed to dim light for 1 week and subsequently full light for 2 weeks. Growth recovery assessment of treated PLBs was then conducted after 4 weeks of incubation in VW10 medium. Growth regeneration percentage of cryopreserved and non-cryopreserved PLBs was recorded based on the ability of treated PLBs to regenerate into new shoots or form new PLBs.

For cryopreservation treatment supplemented with ascorbic acid, all working solutions and media containing exogenous ascorbic acid were prepared devoid of ethylenediaminetetraacetic acid ferric sodium (Fe Na-EDTA) and plant growth regulators. Ascorbic acid (HmbG Chemicals, Barcelona, Spain) stock solution was prepared fresh and filter sterilized (0.45 μm Sartorius Minisart ® NML syringe filter) before added into working solutions. All working solutions were adjusted to pH 5.0 after adding ascorbic acid stock solution. Ascorbic acid with the concentration of 0.284, 0.568 and 0.852 mM (50, 100 and 150 mg/L) based on Uchendu et al. [14] was incorporated into all 4 critical steps of droplet-vitrification cryopreservation: preculture (0.2 M sucrose for 3 days), loading solution (20 min), unloading solution (20 min) and growth recovery media (VW10).

### 2.3 Enzyme extraction for total soluble protein and antioxidant enzyme activities assessment

Enzyme extraction of PLBs was conducted at various cryopreservation stages based on the modified protocols of Monnet et al. [26] and Poobathy et al. [27]. The selected cryopreservation stages are: Preculture, loading, PVS2, after thawing, unloading and growth recovery after one day for both cryopreserved (+) and non-cryopreserved (-) PLBs. Enzyme extraction of PLBs was repeated for all cryopreservation stages in the experiments supplemented with various concentrations of ascorbic acid (50, 100 and 150 mg/L). After each and every cryopreservation stages, PLBs that weighed 100 ± 5 mg were homogenized in 1 mL pre-chilled plant extraction buffer containing 100 mM potassium phosphate buffer (pH 7.8), 2 mM EDTA (Sigma Aldrich, USA) and 2% polyvinylpyrrolidone (PVP) (Fluka Chemie, Switzerland). The homogenate was centrifuged at 12,000 rpm for 20 min at 4 °C (UNIVERSAL 320 R, Hettich zentrifugen, Germany). The supernatant was stored in -40 °C freezer (Sanyo Biomedical Freezer, MDF-U5411, Japan) until further experiments such as Bradford assay, superoxide dismutase (SOD), catalase (CAT) and ascorbate peroxidase (APX) assay.

### 2.4 Determination of total soluble protein content

Total soluble protein of all enzyme extracts was determined by using the Bradford method [28].

### 2.5 Superoxide dismutase (SOD) assay

SOD activity of PLBs at various cryopreservation stages was determined according to a modified method of Poobathy et al. [27]. The total reaction mixture (3 mL) composed of 50 mM potassium phosphate buffer (pH 7.8), 9.9 mM l-methionine, 57 μM nitroblue tetrazolium 2-hydrochloride (NBT-2HCl) and 100 μL plant extract. Riboflavin (1.3 μM) was added last to the reaction mixture. The final result was presented as enzyme units per mg (U/mg) protein following the formulations demonstrated by Poobathy et al. [27].

### 2.6 Catalase (CAT) assay

CAT assay of plant enzyme extract was adapted from Monnet et al. [26] and Poobathy et al. [27]. The reaction mixture (3 mL in total) contained 100 mM potassium phosphate buffer (pH 7.0), 100 μL plant enzyme extract and 6 mM H_2_O_2_ which was added at the final stage to initiate the reaction. The calculation of CAT activity in U/mg protein was performed according to the formula derived from Flocco and Giulietti [29].

### 2.7 Ascorbate peroxidase (APX) assay

APX assay was estimated following the modified methods of Nakano and Asada [30] and Elavarthi and Martin [31]. The three (3) mL assay mixture contained 50 mM potassium phosphate buffer (pH 7.0), 0.5 mM ascorbate, 0.5 mM H_2_O_2_ and 100 μL plant enzyme extract. Extinction coefficient of reduced ascorbate (2.8 mM^−1^ cm^−1^) was used to calculate the enzyme activity that was expressed as U/mg protein following the formula used for the calculation of CAT activity.

### 2.8 Experimental designs and statistical analyses

Experiments were organized according to a randomized complete block design. For growth recovery assessment of PLBs supplemented with different concentrations of ascorbic acid, each experimental condition composed of three (3) replicates with each replicate containing 10 PLBs. The experiment was repeated once (total six (6) replicates, 60 sample size for each treatment). Each biochemical profile (Total soluble protein, SOD, CAT and APX assay) analysis composed of three (3) replicates with each containing enzyme extraction from 100 ± 5 mg of PLBs per treatment. Two-way ANOVA was conducted to determine the significance of interaction effect of ascorbic acid concentrations with different cryopreservation stages on biochemical profile at significance level p < 0.05. Biochemical profile among all the treatments was compared and analyzed by using one-way ANOVA followed by Duncan Multiple Range post hoc test. Mean values were compared at significant level of p < 0.05. Pearson correlation analysis was conducted to determine the correlation between biochemical profile and growth regeneration percentage of the cryopreserved PLBs at p < 0.05. All the statistical analyses were conducted by using SPSS version 24.

### 2.9 RAPD and SCoT analyses

DNA extraction of PLBs was performed following the manufacturing protocol of Wizard® Genomic DNA purification kit (Promega, USA). The plant DNA was extracted from 3 types of PLBs under different treatment: Control PLBs selected from 4 weeks old cultures, PLBs regenerated from optimized droplet-vitrification cryopreservation technique with the highest regeneration percentage (5%) for both cryopreserved (+LN) and non-cryopreserved PLBs (-LN). All three types of plant DNA samples were screened with 60 decamer RAPD primers (OPC, OPK and OPU series) based on the reference from Lim et al. [32] and Manners et al. [33] and 36 SCoT primers designed by Collard and Mackill [21]. The 20 μL reaction mixture for RAPD and SCoT analysis consisted of 50 ng plant DNA template, 1 × PCR buffer, 1.5 mM magnesium chloride (MgCl_2_), 200 μM deoxyribonucleotide triphosphate (dNTP) mix, 1 unit *Taq* DNA polymerase (EconoTaq®, separate MgCl_2_), 1 μM primer, and autoclaved deionized water were amplified in T100™ Thermal Cycler (Bio-Rad Laboratories, Inc., USA). The PCR amplification conditions for RAPD primers were programmed at initial denaturation at 94 °C for 4 min, followed by 40 cycles of denaturation at 94 °C for 1 min, annealing at each primer’s optimized annealing temperature (Ta) for 1 min (Table 1), extension for 72 °C for 1 min; then a final extension of 72 °C for 10 min. For SCoT primers, the PCR amplification were programmed at 94 °C for 3 min; followed by 40 cycles of denaturation at 94 °C for 1 min, annealing at each primer’s optimized Ta (Table 1) for 1 min, extension for 72 °C for 2 min; then a final extension of 72 °C for 10 min.

**Table 1:** List of RAPD and SCoT primers selected for genetic stability assessment of regenerated cryopreserved PLBs.

Primers	Sequence (5’-3’)	GC content (%)	Tm (°C)	Ta (°C)
**RAPD**				
OPK-02	GTCTCCGCAA	60	35.2	32.0
OPK-10	GTGCAACGTG	60	35.2	32.0
OPK-14	CCCGCTACAC	70	36.9	33.8
OPK-16	GAGCGTCGAA	60	35.6	32.6
OPU-01	ACGGACGTCA	60	36.8	33.8
OPU-02	CTGAGGTCTC	60	36.8	33.8
OPC-15	GACGGATCAG	70	31.1	30.1

**SCoT**				
S5	CAACAATGGCTACCACGA	50	52.6	49.6
S6	CAACAATGGCTACCACGC	56	54.4	51.4
S9a	CAACAATGGCTACCAGCA	50	52.9	49.9
S9b	CAACAATGGCTACCAGCC	56	52.9	49.9
S17	ACCATGGCTACCACCGAG	61	57.1	54.1
S28	CCATGGCTACCACCGCCA	67	60.7	57.4
S31	CCATGGCTACCACCGCCT	67	60.4	57.4
S32	CCATGGCTACCACCGCAC	67	59.1	55.0
S33	CCATGGCTACCACCGCAG	67	58.9	55.0
S34	ACCATGGCTACCACCGCA	61	59.7	55.0
S35	CATGGCTACCACCGGCCC	72	61.7	55.0

*Note*: The melting temperature (Tm) of primers is given by respective manufacturer which are Integrated DNA Technologies (IDT) for OPK and OPC series and Vivantis Technologies for OPU series.

The amplified PCR products were separated using Mini-Sub® Cell GT Horizontal Electrophoresis System (Bio-Rad Laboratories, Inc., USA) filled with 0.5 × TBE buffer on 1.5 % (w/v) agarose gel (7 cm × 10 cm) prepared in 1 × Tris-Borate-EDTA (TBE) buffer and stained with RedSafe™ nucleic acid staining solution (iNtRON Biotechnology, South Korea) for visualization. Selection of primers for both RAPD and SCoT-PCR analysis for the assessment of genetic stability was based on the ability of the primers to produce reproducible and clear bands. All primers that produced bands were repeated at least twice to ensure the reproducibility. The selected RAPD and SCoT primers that used for further genetic stability assessments were listed in Table 1 with their respective melting temperature (Tm) and Ta.

## 3 Results and discussions

With the support of many successful examples such as blackberry [14], *Nephelium ramboutan-ake* [17] and kiwifruit [34], implementation of exogenous antioxidant was thought to be an essential step to improve post-cryopreservation regeneration. Even though in the absence of LN exposure, cryopreservation process exposed PLBs to high oxidative stress during explants excision, massive dehydration treatment, change of temperature and physical injury during manipulation throughout cryopreservation procedure [14]. Particularly for cryostorage of tropical plant species which are lack of tolerance to low temperature, it may further increase free radicals production and cellular damage [35], and this is also the reason why exogenous ascorbic acid was included in all four critical steps of cryopreservation in the present studies. Research demonstrated by Mathew et al. [34] also confirmed that supplementation of 0.4 mM in all media throughout the cryopreservation protocol produced 40 % post-cryo regeneration. However, results in current study were completely opposite, as no improvement of regrowth in cryopreserved PLBs was observed when exogenous ascorbic acid was added throughout cryopreservation (Table 2). Moreover, it was notable that the incorporation of ascorbic acid reduced the growth regeneration percentage of cryopreserved PLBs. It was found that in the absence of ascorbic acid, the growth regeneration percentage of cryopreserved PLBs was recorded at 5.00 %, which was the highest among the other ascorbic acid concentrations tested (Fig. 1C). On the other hand exogenous ascorbic acid did not significantly affect growth regeneration percentage of non-cryopreserved PLBs, regardless of the concentrations used. Similarly, it has been reported that no growth recovery was observed in cryopreserved blackberry shoot tips when ascorbic acid were added in all four critical steps of cryopreservation procedure and callus were formed on treated shoot tips [14]. High concentrations of antioxidant will despite act as pro-oxidants disrupt the redox balance and beneficial ROS required for optimal cellular functioning to support plant growth and development, in turn leads to cellular dysfunction [36]. Moreover, Qian et al. [37] reported that large accumulation of ROS and inhibition of growth of *Arabidopsis thalania* seedlings was observed with exogenous ascorbic acid tested at 2 mM and 8 mM, indicating that exogenous ascorbic acid would also act as a stress factor that affects plant growth.

**Table 2:** The effect of different ascorbic acid concentrations (mg/L) on the growth regeneration percentage of non-cryopreserved (-LN) and cryopreserved (+LN) PLBs.

Ascorbic acid (mg/L)	Growth Regeneration Percentage (%)	
	-LN (control)	+LN
0	95.000 ± 2.236	5.000 ± 2.236^a^
50	98.333 ± 1.667	1.667 ± 1.667^ab^
100	98.333 ± 1.667	0.000 ± 0.000^b^
150	96.667 ± 2.108	0.000 ± 0.000^b^

*Mean values denoted with different letters within the same column indicates statistically significant at *p* < 0.05 followed by Duncan Multiple Range Test. (*LN*: Liquid nitrogen).

There was a significant interaction effect between ascorbic acid concentrations and different cryopreservation stages on the production of protein (F_21,64_ = 6.632, *p* < 0.001), SOD and APX activities (F_21,63_ = 4.505, *p* < 0.001 and F_21,64_ = 4.093, *p* < 0.001 respectively), as shown in Table 3. However, the interaction effect between the two independent variables was not significant in inducing the production of CAT (F_21,64_ = 1.336, *p* = 0.187). In other words, the incorporation of different ascorbic acid concentrations during different cryopreservation stages would strongly affect protein, SOD and APX contents. Similarly, exogenous ascorbic acid has a significant inhibitory effect of on SOD, CAT and APX activities of *Arabidopsis* seedlings due to the inhibition of antioxidant genes transcription [37]. Ascorbic acid is a strong antioxidant that scavenges ROS non-enzymatically. Supplementation of excess ascorbic acid may disrupt the homeostatic balance between antioxidant enzymes and ROS that is simultaneously function as signaling molecules to activate plant’s own defense mechanism. Elimination of ROS by ascorbic acid has decrease the need of the plants to elevate antioxidant enzyme activities [38].

**Table 3:** The effects of different ascorbic acid concentrations (mg/L) on biochemical profile of the PLBs during different cryopreservation stages.

Ascorbic acid (mg/L)	Cryo-Stages	Protein (mg/mL)	CAT (U/mg protein)	SOD (U/mg protein)	APX (U/mg protein)
0	Preculture	0.283 ± 0.060^b^	8.205 ± 2.511^b^	4.730 ± 1.145^bcdefg^	7.877 ± 2.259^cd^
0	Loading	0.368 ± 0.017^a^	5.041 ± 1.406^b^	2.426 ± 0.165^defg^	7.473 ± 0.922^cd^
0	PVS 2	0.384 ± 0.011^a^	5.520 ± 1.343^b^	3.057 ± 0.111^bcdefg^	3.573 ± 1.076^cd^
0	Thawing	0.233 ± 0.015^bc^	54.950 ± 25.857^b^	2.282 ± 0.321^defg^	12.941 ± 2.103^cd^
0	+ Unload	0.225 ± 0.019 ^bcd^	57.083 ± 27.882^b^	2.820 ± 0.359^bcdefg^	10.491 ± 1.606^cd^
0	+ GR	0.020 ± 0.000^ij^	320.730 ± 113.872^a^	6.304 ± 2.360^bcdef^	79.751 ± 20.934^a^
0	- Unload	0.235 ± 0.009^bc^	17.693 ± 4.823^b^	3.105 ± 0.969^bcdefg^	10.098 ± 3.452^cd^
0	- GR	0.152 ± 0.037^f^	20.699 ± 7.315^b^	4.339 ± 1.630^bcdefg^	20.730 ± 3.407^bcd^
50	Preculture	0.123 ± 0.007^fgh^	89.775 ± 27.215^b^	5.134 ± 0.896^bcdefg^	20.613 ± 4.974^bcd^
50	Loading	0.159 ± 0.016^def^	56.019 ± 29.991^b^	3.343 ± 0.053^bcdefg^	10.291 ± 1.755^cd^
50	PVS 2	0.148 ± 0.025^f^	35.179 ± 13.110^b^	4.523 ± 0.865^bcdefg^	11.307 ± 4.738^cd^
50	Thawing	0.136 ± 0.015^fg^	53.635 ± 19.793^b^	3.176 ± 0.933^bcdefg^	12.870 ± 3.358^cd^
50	+ Unload	0.079 ± 0.009^ghi^	92.572 ± 29.982^b^	6.723 ± 1.008^bcd^	21.610 ± 1.570^bcd^
50	+ GR	0.010 ± 0.000^j^	327.169 ± 185.803^a^	18.445 ± 0.807^a^	38.910 ± 20.448^b^
50	- Unload	0.182 ± 0.004^cdef^	62.626 ± 17.332^b^	3.430 ± 0.505^bcdefg^	5.349 ± 1.104^cd^
50	- GR	0.118 ± 0.008^fgh^	47.774 ± 9.799^b^	0.866 ± 0.351^g^	14.145 ± 2.715^cd^
100	Preculture	0.132 ± 0.022^fg^	36.389 ± 17.227^b^	1.916 ± 0.949^fg^	16.126 ± 4.681^cd^
100	Loading	0.131 ± 0.023^fg^	60.486 ± 31.679^b^	5.594 ± 0.317^bcdefg^	14.641 ± 6.347^cd^
100	PVS 2	0.131 ± 0.031^fg^	15.819 ± 6.726^b^	6.553 ± 3.136^bcde^	10.746 ± 4.892^cd^
100	Thawing	0.173 ± 0.014^cdef^	32.925 ± 6.331^b^	4.875 ± 1.338^bcdefg^	8.845 ± 1.451^cd^
100	+ Unload	0.134 ± 0.014^fg^	38.143 ± 14.994^b^	6.061 ± 0.606^bcdef^	12.346 ± 1.410^cd^
100	+ GR	0.031 ± 0.004^ij^	74.927 ± 27.448^b^	5.082 ± 3.541^bcdefg^	12.359 ± 3.080^cd^
100	- Unload	0.176 ± 0.006^cdef^	45.083 ± 7.526^b^	3.696 ± 0.366^bcdefg^	6.416 ± 1.481^cd^
100	- GR	0.222 ± 0.053^bcde^	43.913 ± 27.042^b^	2.106 ± 0.271^efg^	8.801 ± 4.348^cd^
150	Preculture	0.133 ± 0.015^fg^	63.270 ± 24.834^b^	6.959 ± 2.744^bc^	14.484 ± 0.621^cd^
150	Loading	0.165 ± 0.010^def^	52.939 ± 25.476^b^	4.659 ± 0.745^bcdefg^	11.541 ± 2.699^cd^
150	PVS 2	0.181 ± 0.012^cdef^	70.164 ± 26.647^b^	2.565 ± 0.594^cdefg^	7.896 ± 2.999^cd^
150	Thawing	0.157 ± 0.014^ef^	53.276 ± 13.833^b^	4.494 ± 0.287^bcdefg^	7.052 ± 1.845^cd^
150	+ Unload	0.143 ± 0.007^fg^	37.563 ± 4.833^b^	4.710 ± 0.180^bcdefg^	13.681 ± 0.293^cd^
150	+ GR	0.045 ± 0.004^ij^	150.845 ± 40.970^b^	3.972 ± 0.955^bcdefg^	13.146 ± 2.254^cd^
150	- Unload	0.182 ± 0.005^cdef^	32.514 ± 0.826^b^	3.107 ± 0.498^bcdefg^	6.721 ± 2.080^cd^
150	- GR	0.063 ± 0.009^hij^	120.462 ± 32.796^b^	7.168 ± 0.934^b^	24.779 ± 5.595^bc^
Ascorbic acid Df (n-1) = 3		55.716 ***	2.014	3.464 *	3.045 *
Cryo stages Df (n-1) = 7		33.796 ***	7.934 ***	6.095 ***	9.395 ***
Ascorbic acid x Cryo stage Df (n-1) = 21		6.632 ***	1.336	4.505 ***	4.093 ***

Mean values denoted with different letters within the same column indicates statistically significant at *p* < 0.05 followed by Duncan Multiple Range Test. F-values denoted with asterisks were significant at different significance levels: * *p* < 0.05; *** *p* < 0.001 followed by two-way ANOVA analysis.

Accumulation of total soluble protein were observed throughout dehydration treatment and cold acclimation in conjunction with PVS2 dehydration at 0 °C for PLBs without ascorbic acid supplementation throughout cryopreservation procedure (Table 3). The induction of protein content could be due to an up-regulation of stress-responsive protein to protect plant cells. Accumulation of dehydrin, which is a subset of late-embryogenesis abundant (LEA) protein, has been found in asparagus embryogenic cells in conjunction with high sucrose incubation before cryopreservation, and this could be related to increase of freezing tolerance in plant cells [39]. On the other hand, a drastic reduction of total soluble protein in PLBs was observed at growth recovery stage after storage in LN and this phenomenon was accompanied by freezing injuries due to high ROS production that reacts and degrades plant proteins. It was also noted that there were no significant differences among all the protein contents at this stage when the cryopreserved PLBs were exposed to different concentrations of ascorbic acid. Rinalducci et al. [40] highlighted that proteins in plants are the most abundant macromolecules oxidized by ROS which comprised of approximately 68 % of the oxidized molecules in cell.

Antioxidant enzymes such as SOD, CAT and APX are part of the protein products from stress tolerance mechanism in plants. Scavenging activity of plant antioxidants at high level was known to enhance abiotic stress tolerance in plants [41]. SOD only catalyzes the dismutation of superoxide radicals to H_2_O_2_, while the ability to reduce osmotic stress is largely dependent on the ability of other antioxidants such as CAT and APX to detoxify the ROS product of SOD enzymes. From the results, the highest CAT content was recorded at growth recovery (+GR) stage for both cryopreservation treatment without exogenously applied ascorbic acid (320.73 U/mg protein) and also cryopreservation exposed to 50 mg/L ascorbic acid (327.17 U/mg protein) (Table 3). On the opposite, there were no significant differences in term of CAT content among all the other ascorbic acid concentrations and cryopreservation stages.

It was found that 50 mg/L ascorbic acid also significantly induced SOD content (18.445 U/mg protein) at growth recovery stages of cryopreserved PLBs (+GR) compared to other cryopreservation stages (Table 3). The drastic increased in SOD and CAT activity of PLBs particularly at growth recovery stage (+GR) with 50 mg/L ascorbic acid supplementation indicated the oxidative burst of superoxide radicals after freeze-thaw treatment. Furthermore, it was known that the induction of CAT activity was due to the action of SOD to produce high level of H_2_O_2_, thus trigger the increases of CAT activity to detoxify excess free radicals. As a result, high level of CAT activity in cryopreserved PLBs at growth recovery stage (+GR) could be correlated with the high SOD activity. Similarly, Poobathy et al. [27] reported that the concurrent increase in CAT activity of the *Dendrobium* sonia-28 PLBs is due to the action of high SOD activity. Low level of CAT activity was unable to cope with high levels of H_2_O_2_ which led to poor survival of cryopreserved *Dendrobium* sonia-28 PLBs [27]. The current studies also revealed the effect of exogenous ascorbic acid at higher concentrations (100 and 150 mg/L) in various cryopreservation stages, where the supplementation has no significant effect in inducing SOD and CAT activity of PLBs during cryopreservation. This phenomenon accelerated the accumulation of ROS in higher amount [16], which in turn contributed to oxidative damage that caused cell death. On the other hand, due to ROS scavenging capability of exogenous ascorbic acid at higher dose, the activation of antioxidant enzyme mechanism in PLBs is not required to detoxify over production of ROS.

In addition, the highest APX activities was recorded in PLBs recovered from cryopreservation treatment (+GR), whilst the lowest APX production (3.573 U/mg protein) was induced during PVS 2 stage without exogenously applied ascorbic acid (Table 3). Willekens et al. [42] reported that deficiency of CAT enzyme in plants trigger the activity of APX and gluthatione peroxidase (GPX), suggesting that the function is to compensate for CAT suppression [43]. However, the finding was opposed to the current studies in cryopreservation of *Aranda* Broga Blue PLBs, as the APX activity was not significantly induced during low CAT activity, regardless the cryopreservation procedure were added with different concentrations of ascorbic acid. In contrast, during control cryopreservation procedure without ascorbic acid, the APX activity pattern was concomitant with CAT activity, where the induction of APX activity was significantly notable at growth recovery stage after cryopreservation (+GR). Study done by Xu et al. [44] confirmed that exogenous ascorbic acid did not influence the antioxidant enzymes activity of the tall fescue including APX activity during water stress. Exogenous ascorbic acid neutralized oxidative stress by directly scavenge ROS in treated plants by the non-enzymatic pathway without involving enzymatic activities [44].

Generally, cryopreserved PLBs at growth recovery stages (+GR) produced significantly high amount of CAT and APX without the supplementation of ascorbic acid, while the highest amount of CAT and SOD were promoted by the incorporation of 50 mg/L ascorbic acid. Linear correlation analysis between APX and growth regeneration percentage of cryopreserved PLBs was found to be significant at *p* < 0.01 (Table 4). The positive Pearson coefficient of 0.872 suggested that APX content is directly proportional to the growth regeneration percentage of the cultures. On the other hand, the correlation between APX content and growth regeneration percentage was not significant in the absence of liquid nitrogen. This study also discovered that linear correlation between protein and growth regeneration percentage of the cryopreserved cultures (Pearson coefficient of -0.481) was not significant at *p* < 0.05. Similarly, CAT and SOD activities of cryopreserved PLBs were found to be of no significant effect on the growth regeneration percentage (Table 4) in accordance to Pearson correlation analysis result. This suggests that the amounts of protein, CAT and SOD do not play significant role in influencing the PLBs regeneration capacity in cryopreservation. Several studies reported that high antioxidant enzyme activities are responsible to improve their cryoprotective mechanism against oxidative burst throughout cryopreservation, which further contributed to higher survival rate. Correlation analysis conducted by Chen et al. [45] indicated that there is a strong relation between high antioxidant enzymes activities including CAT enzymes and indigenous ascorbic acid content, to growth recovery of cryopreserved *Arabidopsis thalania* seedlings. As opposed to present results, protein content and all antioxidant enzyme activities were not correlated to growth recovery of cryopreserved PLBs, except for APX activities. High recovery of cryopreserved PLBs without ascorbic acid supplementation (5%) was highly attributed to increased APX content in growth recovery stage (+GR) (Table 3). CAT and APX enzymes take part in H_2_O_2_ scavenging metabolism and both the enzymes act at different cellular sites through different mechanism. In contrast to CAT enzyme, APX has a higher affinity to H_2_O_2_ because ascorbic acid was required as substrate to catalyze the detoxification process of ROS [42]. Zha et al. [46] mentioned that APX activity of lettuce plants showed greatest response to continuous light exposure among the antioxidant enzymes tested imply that the APX could be the principle antioxidant enzyme for efficient protection against ROS accumulation. In such situation, *Aranda* Broga Blue PLBs may rely largely on APX to control ROS level during cryopreservation, which makes the adjustment of APX an important approach to improve post-cryo recovery.

**Table 4:** Linear correlations (Pearson’s coefﬁcient) between growth regeneration percentage and biochemical profiles of non-cryopreserved (-LN) and cryopreserved (+LN) PLBs.

	Growth regeneration percentage	Protein	CAT	SOD	APX
**-LN**					
Growth regeneration percentage	1.000	0.058	0.214	−0.114	0.087
Protein		1.000	−0.628*	−0.497	−0.765**
CAT			1.000	0.446	0.328
SOD				1.000	0.582*
APX					1.000

**+LN**					
Growth regeneration percentage	1.000	−0.481	0.153	0.197	0.872**
Protein		1.000	−0.444	−0.708*	−0.474
CAT			1.000	0.466	0.228
SOD				1.000	0.074
APX					1.000

Values denoted with asterisks were statistically significant at with different levels of signiﬁcance: * *p* < 0.05; ** *p* < 0.01. (*LN*: Liquid nitrogen).

On the other hand, studies conducted by Distelbarth et al. [47] emphasized that although oxidative disturbance of four accessions of *Arabidopsis* occurred during cold acclimation, however, there was a lack of correlation of ROS reacted to freezing tolerance. Thus, the key role for adjustment of ROS defense in response to cryopreservation treatment may not be beneficial as the response of antioxidant activities to various plant stress are considered species or variant dependent. Many cryobiologist has been diverted to add anti-stress compound in cryopreservation protocol due to enormous promising results, but this may not be advantages for plants that require activation of natural antioxidant enzyme activities to combat plant stress. For tropical plants which are cold susceptible, antioxidant enzymes activities may not be part of the protective mechanism against cold or freezing stress. At least from present studies, we speculated that high water content in PLBs could still be the main hurdle that cause low growth recovery of cryopreserved PLBs, as supplementation of ascorbic acid did not accelerated the growth recovery. On top of that, it has been confirmed that no correlation between antioxidant enzyme activities and growth recovery of cryopreserved PLBs was observed besides APX activities. The application of exogenous antioxidants in suitable cryopreservation stages needs to be carefully evaluated due to the interaction effect of ascorbic acid and cryopreservation that may influences antioxidant activities which will further disrupt the complex protective mechanism of plants. It is suggested that natural antioxidants could be a better alternatives compared to synthetic compounds due to its multifunctional activities that is vital in controlling oxidative damage [16].

**Fig. 2:**
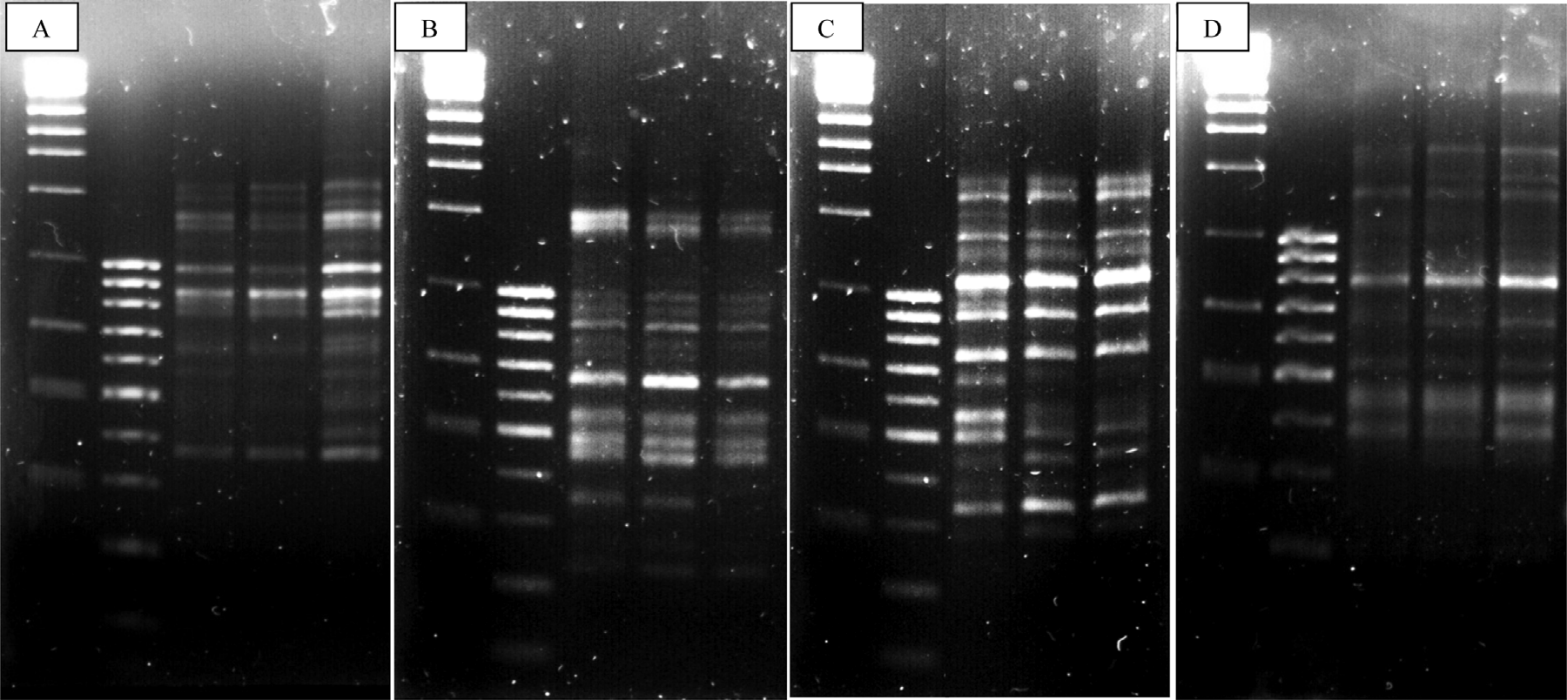
**Banding profile of regenerated cryopreserved (+LN) and non-cryopreserved explants (-LN) compared to *in vitro* control PLBs (UC) using RAPD marker with OPK-02 (A), OPK-10 (B), OPK-14 (C) and OPK-16 (D) primers.** Lane 1 = 1 kb ladder; Lane 2 = 100bp ladder; Lane 3= Untreated control (UC); Lane 4= non-cryopreserved explants (-LN); Lane 5= cryopreserved explants (+LN).

**Fig. 3:**
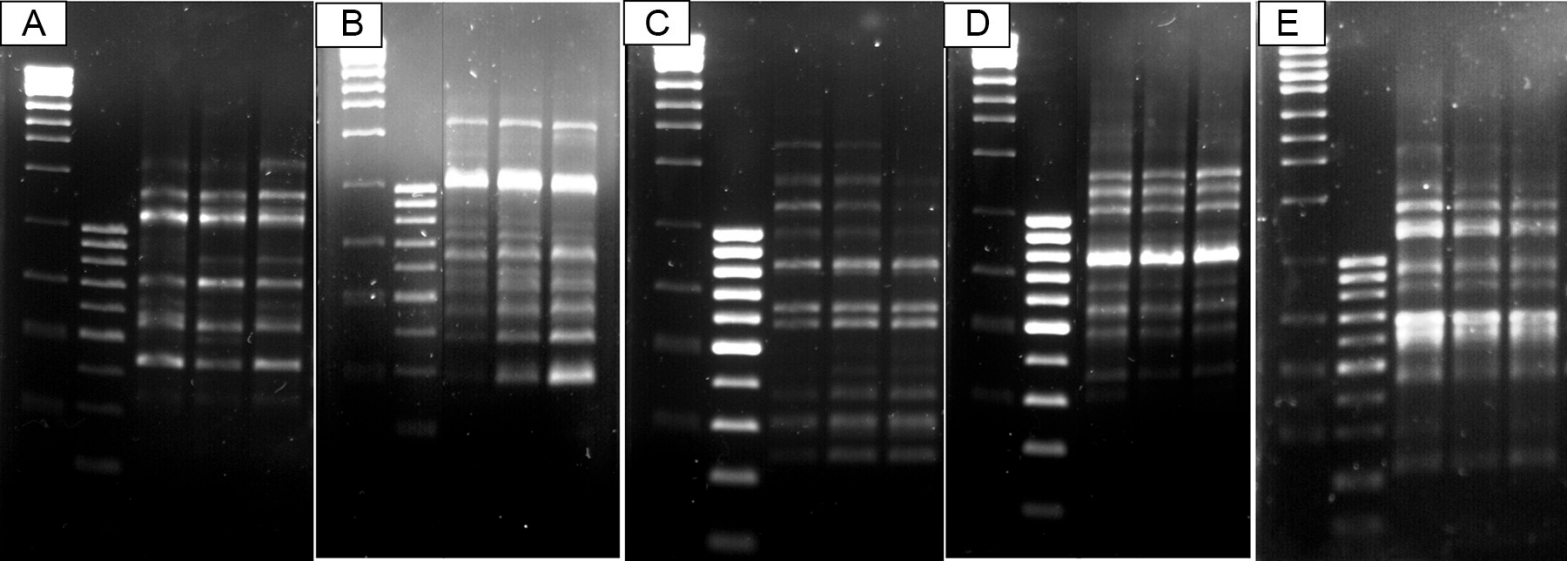
**Banding profile of regenerated cryopreserved (+LN) and non-cryopreserved explants (-LN) compared to *in vitro* control PLBs (UC) using SCoT marker with S9a (A), S9b (B), S33 (C), S34 (D) and S35 (E) primers.** Lane 1 = 1 kb ladder; Lane 2 = 100bp ladder; Lane 3= Untreated control (UC); Lane 4= non-cryopreserved explants (-LN); Lane 5= cryopreserved explants (+LN).

Based on preliminary screening using 60 RAPD decamer primers, only 7 primers (OPK-02, OPK-10, OPK-14, OPK-16, OPU-01, OPU-02 and OPC-15) generated reproducible, unambiguous and clear bands (Table 5). Number of bands produced in each primer ranging from 6 (OPK-16) to 13 (OPK-14) while the size of bands produced were range from 200 to 2000 kb (Fig. 2A-D). With 36 primers tested, initial screening of SCoT primers showed that 11 primers generated clear and reproducible SCoT profile for further genetic stability evaluation. These SCoT primers including S5, S6, S9a, S9b, S17, S28, S31, S32, S33, S34 and S35 primers (Table 5). Each primer produced different number of clear and reproducible bands varied from 6 (S32) to 11 (S6, S31 and S33) bands and band sizes produced were range from 200 to 1800 kb (Fig. 3A-E). There were no polymorphic bands detected in all the selected RAPD and SCoT primers, as regenerants of cryopreserved and non-cryopreserved PLBs produce the same banding pattern compared to untreated control, with the same number and sizes of bands generated in all three treatments.

**Table 5:** List of reproducible RAPD and SCoT markers and the profile generated in untreated control (UC), regenerants from non-cryopreserved (-LN) and cryopreserved (+LN) PLBs.

Primers	Number of amplified bands			Number of polymorphic bands	Number of monomorphic bands	% polymorphism	Band Sizes (bp)
	UC	-LN	+LN				
**RAPD**							
OPK-02	10	10	10	0	10	0	300−1400
OPK-10	11	11	11	0	11	0	200−1500
OPK-14	13	13	13	0	13	0	300−2000
OPK-16	6	6	6	0	6	0	200−1300
OPU-01	8	8	8	0	8	0	400−1300
OPU-02	7	7	7	0	7	0	400−1100
OPC-15	8	8	8	0	8	0	200−1000
**Total**	63	63	63	0	63	0	
**Mean**	9	9	9	0	9	0	

**SCoT**							
S5	10	10	10	0	10	0	300−1800
S6	11	11	11	0	11	0	400−1400
S9a	8	8	8	0	8	0	400−1600
S9b	10	10	10	0	10	0	300−1600
S17	7	7	7	0	7	0	300−1000
S28	7	7	7	0	7	0	400−1800
S31	11	11	11	0	11	0	200−1400
S32	6	6	6	0	6	0	400−1200
S33	11	11	11	0	11	0	200−1400
S34	8	8	8	0	8	0	300−1300
S35	9	9	9	0	9	0	200−1400
**Total**	98	98	98	0	98	0	
**Mean**	8.9	8.9	8.9	0	8.9	0

This suggested that the developed droplet-vitrification cryopreservation protocol in previous studies by Khor et al. [2] is a reliable long term conservation method for *Aranda* Broga Blue orchid PLBs that retain its genetic stability during cryo-storage. Similarly, there are numerous evidence showed that cryopreservation is the most promising conservation method for the maintenance of plant genetic resources, as many cryopreserved explants showed no genetic variation after retrieved from LN based on various molecular analysis such as *Vanda Coerulea* protocorms [48], *Chrysanthemum morifolium* shoot tips [49], oil palm (*Elaeis guineensis Jacq.*) polyembryoids [50] and Argyranthmum shoot tips [51]. In addition, identical banding profile of explants gave rise from non-cryopreserved PLBs compared to untreated control indicated the reliability of sequential cryopreservation treatment that caused only minimal cryo-injury to treated explants, which prevent cryo-injury induced genetic variation in treated explants prior LN storage. Genetic variation could occur during cryopreservation procedure itself, while ultra-low temperature of LN is not the main factor that induced genetic variation during cryopreservation [52,53]. Thus, the choice of suitable treatment which prevents genetic instability of regenerated explants is the main concern during development of cryopreservation procedure for a new plant species.

RAPD profile only reveals the screening of a very small part of the whole plant genome. Thus, validation of generated RAPD profile is required and this can be done by using different molecular marker system. As a consequence, it was suggested that more than one marker system can be utilized to crosscheck and validate the genetic stability of regenerants recovered from cryopreservation treatment. As the amplified region of different marker system varied, it provides a more reliable result of genetic stability studies [[54], [55], [56]].

## 4 Conclusion

The present studies showed that the addition of exogenous ascorbic acid in cryopreservation protocols did not improve growth recovery of *Aranda* Broga Blue PLBs. This study has proven that ascorbic acid could act as a double edge sword as it may disturb natural protective mechanism of plants prior cryopreservation through the assessment of various antioxidant enzyme activities. It is suggested that APX activities were highly associate with growth recovery of cryopreserved PLBs, but not the other antioxidant enzymes tested. Through better understanding of the factors that contributes to growth recovery of cryopreserved explants, this will facilitate the development of suitable cryopreservation methodologies for various plant species.

## Author’s contribution statement

Soo Ping Khor and Sreeramanan Subramaniam designed and performed the experiments, processed experimental data and wrote the manuscript. Lit Chow Yeow performed the statistical analyses and assist in manuscript writing. Ranjetta Poobathy assists in results interpretation. Sreeramanan Subramaniam, Bee Lynn Chew, and Rahmad Zakaria supervised the project. All authors discuss the results and contributed to the manuscript.

## Declaration of Competing Interest

None.
